# Optimized cultivation of *Campylobacter concisus* from gut mucosal biopsies in inflammatory bowel disease

**DOI:** 10.1186/s13099-016-0111-7

**Published:** 2016-06-01

**Authors:** Karina Frahm Kirk, Hans Linde Nielsen, Ole Thorlacius-Ussing, Henrik Nielsen

**Affiliations:** Department of Infectious Diseases, Aalborg University Hospital, Aalborg, Denmark; Department of Clinical Medicine, Aalborg University, Aalborg, Denmark; Department of Clinical Microbiology, Aalborg University Hospital, Aalborg, Denmark; Department of Gastrointestinal Surgery, Aalborg University Hospital, Aalborg, Denmark

**Keywords:** *Campylobacter concisus*, Inflammatory bowel disease, Crohn’s disease, Ulcerative colitis, IPAA, Pouch

## Abstract

**Background:**

*Campylobacter concisus* is a commensal of the human oral flora that has been linked to prolonged diarrhea and inflammatory bowel disease (IBD). It has been detected more often from intestinal biopsies in patients with IBD compared to healthy controls using PCR-based techniques, whereas the number of *C. concisus* culture-positive biopsies in previous studies has been very limited. Determining the rate of viable isolates present in the gut mucosa is of great importance when evaluating the role in different disease presentations. We therefore investigated a novel two-step cultivation procedure combining anaerobic and microaerobic incubation from several gut mucosal sites to improve isolate yield, and compared this to PCR results, from IBD patients and healthy controls.

**Results:**

Cultivation with the novel two-step procedure yielded a higher rate of *C. concisus* isolates from mucosal biopsies than previously reported by other methods. From 52 IBD patients, 52/245 (21 %) biopsies were culture positive for *C. concisus*, while 121/245 (49 %) of biopsies were PCR positive. For 26 healthy controls, the numbers were 23/182 (13 %) and 66/182 (36 %), respectively (p < 0.001). The rate of cultivation and PCR detection was higher for IBD patients compared to healthy controls (p = 0.021, p = 0.008, respectively).

**Conclusions:**

Patients with IBD had a higher prevalence of *C. concisus* than healthy controls, by both cultivation and PCR detection. We found a higher rate of *C. concisus* isolates from gut mucosal biopsies in both IBD patients and healthy controls than in preceding studies, indicating that colonization of *C. concisus* in the gastrointestinal tract is more extensive than previously assumed.

## Background

The genus *Campylobacter* consists of 26 species, whereof *Campylobacter jejuni* is the leading cause of gastroenteritis in humans worldwide [1]. Meanwhile, mounting evidence has brought attention to the clinical significance of other *Campylobacter* species in gastrointestinal disease, therefore termed emerging *Campylobacter* species [2]. *Campylobacter jejuni, Campylobacter coli* and *Campylobacter lari* are thermophilic, with poultry as the main reservoir and source of infection in humans. In contrast, the optimal growth temperature for many emerging *Campylobacter* species is 37 °C [3] suggesting that mammals, including humans, could serve as ideal reservoir candidates.

Several studies have associated *Campylobacter concisus* to prolonged gastroenteritis, in children as well as adults [4–6]. Furthermore, recent studies have found a high prevalence of *C. concisus* DNA in mucosal biopsies from patients with inflammatory bowel disease (IBD) using PCR-based methods [7–10], whereas cultivation of isolates has been sparse and to date, only few isolates have been recovered. A recent meta-analysis of these previous studies found that *C. concisus* was associated with an increased risk of IBD (OR 3.76, p = 0.006) [11]. Enteric *C. concisus* isolates have been shown to possess pathogenic capacities such as cell bound and secreted hemolytic activity that can facilitate increased intestinal permeability—a pathognomonic finding in IBD [12]. However, a vast difference in pathogenic properties between isolates exist and a study examining the genomes of different enteric *C. concisus* isolates has verified the pronounced diversity of the species [13]. The two major disorders that comprise IBD are Crohn’s disease (CD) and ulcerative colitis (UC). While these disorders are thought similar in etiology, the pathogenesis is very different, with CD affecting the entire gastrointestinal tract and UC only the large intestine [14]. The aim of the present study was to assess whether cultivation of *C. concisus* from gut mucosal tissue could be optimized by incubating in both microaerobic and anaerobic atmospheres to yield a higher number of isolates, and to compare these results to a polymerase chain reaction (PCR) method.

## Methods

In this study, we tested the effect of cultivation in different atmospheres on recovery of emerging *Campylobacter* species from gut mucosal biopsies, collected from 52 adult patients with IBD and 26 healthy controls, and compared the cultivation results to detection by PCR. Twenty-seven of the IBD patients had previously undergone surgery with restorative proctocolectomy and ileal-pouch-anal-anastomosis (IPAA) due to severe UC [15]. All IBD patients had a disease history of at least 1 year, and no study participants had received antibiotics in the month prior to study enrolment. Healthy controls and IBD patients except for those with IPAA had undergone standard bowel preparations prior to the procedure. Patients with IPAA were flushed with saline at the start of the endoscopy. An overview of the study participants is presented in Table 1. The study was approved by the Regional Ethics Committee of Northern Jutland, Denmark (N-20130070), and all study participants provided written consent.

**Table 1:** Disease subgroups, sex, age and number of samples collected from IBD patients and healthy controls (HC)

	IBD			HC
Disease	CD	UC	IPAA	–
Number	9	16	27	26
Sex, male (%)	4 (44)	7 (44)	13 (50)	15 (58)
Age, median (IQR)	42 (31–49)	59 (40–63)	43 (35–50)	57 (49–66)
Samples, median (range)	7 (6–7)	7 (4–7)	3 (3–3)	7 (7–7)

Biopsies were collected from the terminal ileum, cecum, ascending-, transverse-, descending-, sigmoid colon and rectum. From IPAA patients, biopsies were collected from the ileum, and the proximal and distal part of the pouch. Saliva samples from each patient were also collected, and stool samples from all but four IBD patients and one healthy control. Healthy controls were patients undergoing colonoscopy for other indications such as screening for colorectal cancer, or abdominal pain without any symptoms of enteritis. Biopsies were collected with sterile forceps (Medwork, Braun Scandinavia, Denmark), which were discarded after each individual biopsy. Tissue samples were promptly placed in sterile containers with 0.5 ml sterile saline and immediately taken to the laboratory, rendering a transit time of less than 30 min at ambient temperature.

Each biopsy was smeared onto two non-selective 5 % blood agar plates with added yeast extract (SSI Diagnostica, Denmark) using sterile inoculation loops. Cultivation was then carried out as a two-step procedure: Initially, one plate was incubated for 48 h in microaerobic (80 % N_2_, 10 % CO_2_, 5 % H_2_, 6 % O_2_) and the other in anaerobic (80 % N_2_, 10 % CO_2_, 10 % H_2_) conditions, attained using the Anoxomat Mart II system (Mart Microbiology B.V., Netherlands). Following 48 h of incubation, inoculation loops were used to harvest approximately 100 µg bacterial mass by streaking across each agar plate. The bacterial mass was liquefied by addition of 50 µl sterile saline and brief vortexing. Using sterile pipettes, the emulsion was transferred onto two 5 % blood agar plates with added yeast extract (SSI Diagnostica, Denmark) and polycarbonate filters (0.6 µm pore size) (Whatman^®^ Nuclepore™, Sigma-Aldrich, MO, USA), then incubated for 60 min at 37 °C in an ambient atmosphere, using a previously described method [16]. Subsequently, all plates were incubated in microaerobic conditions for a total of 96 h. On daily inspection, colonies resembling *Campylobacter* spp. were subjected to microscopic examination and species specificity was confirmed by MALDI-TOF (BRUKER DALTONIK GmbH, Bremen, Germany) analysis [17]. *Campylobacter concisus* isolates were also subjected to qPCR analysis (Life Technologies, Carlsbad, CA, USA) with the *C. concisus* specific primers targeting the 16S rRNA gene with modifications of the methods of Mahendran et al. [9]. Stool and saliva samples were cultivated by previously described methods [16], and incubated in a microaerobic atmosphere only.

After cultivation, biopsies were prepared for PCR examination by initial disruption of tissue samples using the Tissuelyser II (Qiagen, Hilden, Germany) according to the manufacturer’s instructions. DNA was extracted using an on-board protocol with the NucliSENS^®^ easyMAG^®^ platform (BioMérieux, Marcy-l’Étoile, France). Real-time PCR (Life Technologies, Carlsbad, CA, USA) was then performed with modifications of the methods previously described by Mahendran et al. [9], with initial amplification of bacterial 16S rRNA genes, followed by PCR purification and finally *C. concisus* specific PCR using the primers Conc Fmod and Conc R2 using the conditions described by Watt et al. [18]. Amplified PCR products were visualized on QiAXcel Advanced screen gel for verification of product size (Qiagen, Hilden, Germany).

Data was analysed using Stata 14 (Statacorp LP, Texas, USA). Fischer’s exact test was used for dichotomous variables and McNemar’s test for paired nominal data. A p value < 0.05 was considered statistically significant.

## Results

In total, 427 biopsies were collected from 78 subjects. From gut mucosal biopsies, isolates that grew in both microaerobic and anaerobic atmospheres were collected separately, rendering a total of 99 isolates. Twenty-nine isolates were derived from microaerobic and 22 from anaerobic incubation exclusively (p = 0.40). From 24 biopsies, isolates grew in both atmospheres (Table 2).

**Table 2:** Number of *C. concisus* positive biopsies from gut mucosal tissue, derived from different incubation methods from IBD patients and healthy controls (HC) (p = 0.40)

Incubation method	IBD (%)	HC (%)
Microaerobic only	18 (25)	11 (39)
Anaerobic only	17 (24)	5 (18)
Both	36 (51)	12 (43)
Total	71	28

In all IBD patients, a total of 52/245 (21 %) biopsies were culture positive for *C. concisus*, while 121/245 (49 %) were PCR positive (p < 0.001) For healthy controls, the numbers were 23/182 (13 %) and 66/182 (36 %), respectively (p < 0.001). The rate of cultivation and PCR detection was higher for IBD patients compared to healthy controls (p = 0.021 and p = 0.008, respectively). Interestingly, the rate of *C. concisus* cultivation and PCR detection was significantly higher for IPAA patients compared to other IBD patients (p = 0.0001 and p = 0.0006, respectively), and there was no significant difference between CD and other UC patients. The number of culture and PCR positive biopsies from different disease categories are shown in Table 3.

**Table 3:** The number of *C. concisus* positive biopsies from the total number of biopsies obtained from the different patient groups, by cultivation and PCR

	Cultivation (%)	PCR (%)
CD	9/61 (15)	21/61 (34)
UC	13/103 (13)	47/103 (46)
IPAA	30/81 (37)	53/81 (65)
HC	23/182 (13)	66/182 (36)
Total	75/427 (17.5)	187/427 (44)

**Fig. 1:**
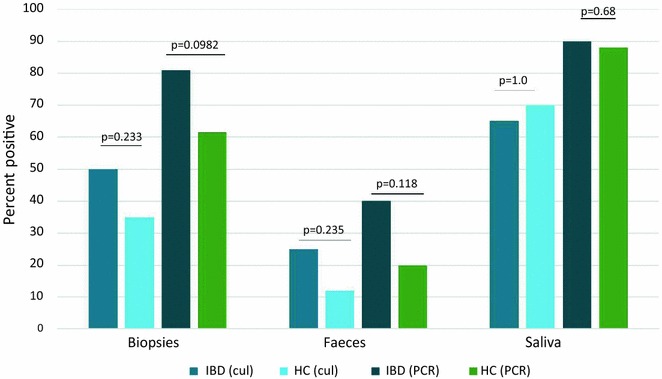
Percentage of *C. concisus* positive individuals in at least one biopsy, faeces and saliva by cultivation (cul) and PCR

From stool samples, 12/48 (25 %) of IBD patients were culture positive, while 19/48 (40 %) were positive by PCR detection. When comparing IPAA patients to other IBD patients, there was no difference in isolation from stool by cultivation (p = 0.18), but a higher detection rate by PCR (p = 0.001). For healthy controls, the numbers were 3/25 (12 %) and 5/25 (20 %) respectively. As expected, *C. concisus* was abundant in saliva samples from both IBD patients and healthy controls, with 34/52 (65 %) IBD and 18/26 (70 %) healthy controls being culture positive, 47/52 (90 %) and 23/26 (88 %) PCR positive, respectively. When assessing the number of positive subjects with at least one positive *C. concisus* biopsy, stool or saliva sample, there was no difference between groups with either cultivation or PCR methods, although there was a trend towards higher PCR detection rates from biopsies and stool in IBD patients compared to healthy controls (Fig. 1). Agar plates were also inspected for *Campylobacter* species other than *C. concisus*. The majority of these were *C. ureolyticus,* with six isolates recovered from six different IBD patients. One of these patients was also positive for *C. concisus.* One IBD patient had *C. curvus* in three different biopsies; another had one *C. showae* isolate in one biopsy. Both of these patients were also positive for *C. concisus*. In the healthy control group, isolates of *C. ureolyticus* and *C. showae* were recovered from four different individuals, whereof two were also positive for *C. concisus.* No thermophilic *Campylobacter* species were isolated from IBD patients or healthy controls.

## Discussion

To our knowledge, this is the first study to address the issue of difficulty in cultivating emerging *Campylobacter* species from mucosal tissue. While PCR methods are more sensitive than cultivation in the detection of *C. concisus* from gut mucosal biopsies, it is possible that some of the DNA detected could be remnants from the oral cavity in which *C. concisus* is abundant. Furthermore, cultivation of isolates is essential as it can contribute information regarding virulence properties and antibiotic susceptibility, providing useful knowledge when exploring a possible association to different clinical illnesses. The cultivation of *C. concisus* from mucosal biopsies is tedious, but the prevalence of viable *C. concisus* is high in both IBD and healthy subjects, indicating that colonization by *C. concisus* is not restricted to the oral cavity. It is plausible that *C. concisus* has capacities for conforming to different environmental settings through genetic and epigenetic variations, much like observed for *C. jejuni* [19]. Alterations in the genome during host infection could then explain the existence of *C. concisus* in different parts of the gastrointestinal tract, possibly also explaining the difference in nutritional requirements observed in the oral cavity, as opposed to the intestine.

Most *Campylobacter* species metabolize amino acids or tricarboxylic acid cycle intermediates for growth in microaerobic conditions. Six species, *C. concisus*, *C. curvus, C. gracilis, C. rectus, C. showae* and *C. ureolyticus* have been described to require hydrogen or formate as an electron donor in this process [3]. Recently, Lee et al. examined the growth of *C. concisus* in different atmospheres and observed that oral and enteric *C. concisus* isolates grew in anaerobic conditions without the presence of H_2_, formate or fumarate, although the presence of H_2_ increased growth [20].

Different methods for attaining microaerobic and anaerobic atmospheres for cultivation of *Campylobacter* species are available; most commonly the gas generating sachets or automated gas delivery systems. In previous studies, no significant difference in cultivation rates between the two systems has been observed, although it appears that colony size may increase when using the automated gas delivery system [21–23]. We found that combining initial anaerobic cultivation of mucosal tissue with traditional microaerobic cultivation yielded a high recovery rate for *C. concisus*. Invasive strains of *C. concisus* have been shown to retain capacity for intracellular survival in host cells [24], possibly making growth in anaerobic atmospheres more favorable. It is remarkable that for IBD patients almost equal numbers of isolates grew in microaerobic and anaerobic environments, while the majority of isolates from healthy individuals grew in a microaerobic atmosphere only (Table 1), which could reflect a different pathogenic feature of IBD isolates. The prevalence of *C. concisus* in faecal samples from diarrheic patients has previously been found to be almost as high as for *C. jejuni/C. coli* [25]. In this study, we also found a high prevalence of *C. concisus* in faeces, although not as high as in gut mucosal biopsies. This may be because several biopsies were attained from each individual, and because incubation was performed in two different environments. Stool and saliva samples were incubated in a microaerobic atmosphere only and as such, isolate yield may be inadequate and therefore underestimate the actual prevalence of viable *C. concisus* in these samples.

It cannot be ruled out, that the increased cultivation rate can be explained by simply cultivating on more than one agar plate, regardless of the incubation method used, and further studies to elucidate genetic differences in isolates recovered from the same site but different environments, are currently undergoing in our laboratory. The percentage of subjects with at least one positive *C. concisus* biopsy was considerably larger in our study compared with previous findings, as was the isolate yield—probably due to the fact, that we examined more biopsies from each individual. While isolate yield was more extensive in IBD patients than healthy controls, there was no difference in the number of persons with at least one positive biopsy. This could indicate that gastrointestinal disease correlates to increased bacterial load or abundance of *C. concisus* in the gastrointestinal tract, rather than only bacterial presence. We also found that UC patients with IPAA had a higher prevalence of *C. concisus* in mucosal biopsies compared to other IBD patients and healthy controls. IPAA surgery is performed on UC patients with medically refractory disease or severe clinical complications. The higher prevalence of *C. concisus* in IPAA patients could be associated to the history of severe inflammation, and the pathogenic potential of such isolates need to be evaluated. Previously, it has been proposed that the procedure for bowel preparations for colonoscopy could influence the isolation rates of *C. concisus*, due to the induction of severe diarrhea that could flush the bacteria from the gut lumen [9]. Patients with IPAA do not undergo standard procedures for bowel preparations with induction of severe diarrhea, but diarrhea is a common clinical feature of their condition, so this is an unlikely explanation of the difference. By optimizing cultivation procedures, this study has shown that *C. concisus* colonization in the gastrointestinal tract is extensive, warranting the need for further studies comparing isolates from IBD patients and healthy controls.

## Conclusions

The rate of *C. concisus* detection by PCR was higher for IBD patients than healthy controls, similar to findings in previous studies. The rate of *C. concisus* isolates recovered by cultivation was higher for IBD patients compared to healthy controls, but there was no difference in the proportion of individuals with at least one positive biopsy. Interestingly, the group of IBD patients with previous IPAA surgery had a higher rate of *C. concisus* by both cultivation and PCR compared to other IBD patients. Combining traditional microaerobic incubation with a two-step procedure that incorporates initial anaerobic cultivation is laborious, but we found that this method facilitates a higher isolation rate of *C. concisus* than by microaerobic incubation exclusively.

## Abbreviations

CD: Crohn’s disease
HC: healthy controls
IBD: inflammatory bowel disease
IPAA: ileal-pouch-anal-anastomosis
PCR: polymerase chain reaction
qPCR: quantitative PCR (real-time PCR)
UC: ulcerative colitis
